# Nettle (*Urtica cannabina* L.) polysaccharides as a novel dietary supplement: enhancing systemic antioxidant status via modulation of the gut–liver axis

**DOI:** 10.3389/fphar.2025.1692189

**Published:** 2025-11-19

**Authors:** Jize Zhang, Qian Zhang, Xiaoqing Zhang, Jiang Qiao, Jingwei Wang, Yang Gao, Ping Dong

**Affiliations:** 1 Key Laboratory for Mode Innovation in Forage Production Efficiency, Ministry of Agriculture and Rural Affairs, Institute of Grassland Research, Chinese Academy of Agricultural Sciences, Hohhot, China; 2 College of Life Sciences, Baicheng Normal University, Baicheng, China; 3 College of Food Science and Engineering, Ocean University of China, Qingdao, China

**Keywords:** *Urtica cannabina* L., polysaccharides, antioxidant, gut–liver axis, gut microbiota, serum metabolites

## Abstract

**Background:**

Nettle (*Urtica cannabina* L.) is a promising traditional food source with great potential in the expanding functional foods market; however, the bioactive potential of its polysaccharides, a major component, remains underexplored as a functional food ingredient. This study evaluated the effects of *U. cannabina* polysaccharides (UP) on gut microbiota modulation and systemic antioxidant activity in healthy mice.

**Methods:**

Mice were fed a basal diet or diets supplemented with low (300 mg/kg) (UPL) and high (600 mg/kg) (UPH) doses of UP for 28 days.

**Results:**

Our findings revealed that UP supplementation, particularly at low doses, significantly improved growth performance (*P* < 0.05), serum lipid profiles (*P* < 0.05), and hepatic and serum antioxidant capacity without inducing liver damage. Notably, UPL treatment reduced malondialdehyde (MDA) levels (*P* < 0.01) and enhanced the activities of superoxide dismutase (SOD), glutathione peroxidase (GSH-PX), catalase (CAT), and total antioxidant capacity (T-AOC) (*P* < 0.05). Sequencing of 16S rRNA indicated that UP supplementation altered gut microbiota composition, particularly by increasing the relative abundance of beneficial genera such as *Parabacteroides* (*P* = 0.0973) and *Dubosiella* (*P* = 0.0648) in the UPL group, which were positively correlated with antioxidant biomarkers. Moreover, UPL treatment elevated levels of short-chain fatty acids (SCFAs), especially acetate and butyrate (*P* < 0.05). Untargeted metabolomics demonstrated that UPL treatment influenced serum metabolic profiles and enriched the bile acid (BA) secretion pathway, with notable increases in deoxycholic and taurocholic acid, suggesting a potential link between gut microbiota, BA metabolism, and host antioxidant status.

**Conclusion:**

These findings indicate that UP could serve as a safe and effective functional dietary supplement capable of improving antioxidant function through gut microbiota modulation and gut–liver axis signaling.

## Introduction

The global demand for functional foods, which offer health benefits beyond basic nutrition, continues to accelerate, driven by increasing consumer awareness of the connection between diet and health (Ataei Nukabadi et al., 2022; Wójcik et al., 2021). This trend has sparked growing interest in plant sources rich in bioactive constituents that are sustainable, multifunctional, and compatible with modern dietary preferences (Kozlowska et al., 2019; Hernández-Pérez et al., 2022; Timm et al., 2023). Among these, medicinal plants stand out due to their diverse phytochemical profiles and long histories of traditional use, offering valuable opportunities for innovation in food and health applications (Belščak-Cvitanović et al., 2015).

Nettle *Urtica cannabina* L., a wild perennial herb of the Urticaceae family, stands out for its rich phytochemical composition and diverse ethnomedicinal uses across continents (Đurović et al., 2020; Kutlu et al., 2020). Traditionally used to manage inflammatory conditions, anemia, and rheumatism, nettle has attracted scientific attention for its antioxidant, antimicrobial, anti-inflammatory, and nutritional properties, which are linked to its abundance of flavonoids, phenolic acids, amino acids, vitamins, and minerals (Bagheri et al., 2021; Fiol et al., 2016). Beyond its pharmacological uses, the aerial parts of nettle play a historical role in traditional diet in both Chinese cuisine and European cooking as a steamed vegetable and as an ingredient in preparations such as soups, omelets, pastas, and cheese making (Marchetti et al., 2018; Mohammadian et al., 2024; Rawat et al., 2020). These applications highlight the dual role of nettle as a dietary and medicinal resource.

While most studies have focused on phenolics and flavonoids (Fattahi et al., 2014; Salim et al., 2020), polysaccharides are in fact the most abundant macromolecular component of nettle. Existing research on nettle polysaccharides is very limited compared to other components. Evidence from other plant-derived polysaccharides has demonstrated that they can influence gut microbiota, support immune and antioxidant functions, and promote metabolic health. These effects are largely mediated through fermentation by gut microbes, which produce short-chain fatty acids (SCFAs) such as acetate, propionate, and butyrate. These metabolites contribute to energy balance, intestinal integrity, and systemic homeostasis (Chassaing et al., 2015; Flint et al., 2012; Kovatcheva-Datchary et al., 2015; Veronese et al., 2018). These findings suggest that nettle polysaccharides may exert similar health-promoting effects. Despite these advances, the biological activity of nettle polysaccharides remains poorly understood. Most research on *Urtica* species has been conducted in pathological or sub-healthy models (Haghshenas et al., 2023; Ilhan et al., 2019), while the effects nettle polysaccharides have as a novel dietary supplement in healthy individuals have not been systematically investigated. Given the critical role of the gut–liver axis in regulating host antioxidant function and metabolic health (Pabst et al., 2023), it is necessary to explore whether UP can beneficially modulate these pathways under physiological conditions.

Hence, we aimed to explore whether the consumption of nettle polysaccharides by healthy subjects has a beneficial effect on the body. In the present study, we hypothesized that alternations in gut microbiota were relevant to the effects on *U. cannabina* polysaccharides (UP) on the host metabolism. Specifically, we assessed how different doses influence gut microbiota composition, SCFA profiles, blood metabolites, and metabolic phenotypes in healthy mice. By linking microbial shifts to systemic metabolic responses, our research not only addresses the knowledge gap regarding the biological role of UP but also provides mechanistic insights into its health-promoting potential in the development of next-generation functional foods and nutraceuticals.

## Materials and methods

### *Urtica cannabina* polysaccharide preparation

The fresh aboveground parts of *U. cannabina* were cultivated and collected in August 2023 at Hohhot, Inner Mongolia, China. The aerial parts of plant samples were air-dried at room temperature and then pulverized into powder with a grinder to pass through a 60-mesh sieve. The preparation procedure of UP was carried out according to Zhang et al. (2013) with slight modifications. The dried plant powder was first treated with petroleum ether to eliminate some small molecular compounds. Then, 200 g of *U. cannabina* powder was extracted with hot water (1:15 w/v) at 60 °C for 4 h. The aqueous extract was collected by filtration with a 0.45-μm filter and concentrated by a rotary evaporator. The concentrated supernatants were precipitated with anhydrous ethanol (ratio 1:4, v/v) for 48 h at 4 °C. The floating precipitation above the ethanol was collected by centrifugation and redissolved in ultrapure water. Then the polysaccharide solution was deproteinated twice with Sevag reagent, decolorized by D101 macroporous resin, and dialyzed and lyophilized by a vacuum evaporator to obtain UP. The total carbohydrate and uronic acid contents of UP were 57.8% and 6.2%, respectively. It is important to note that due to equipment limitations, detailed structural characterization of the UP was not performed. Future studies will aim to include these analyses to provide a more comprehensive physicochemical profile.

### Animal experiment

The animal experiments complied with the National Standard Guideline for Ethical Review of Animal Welfare (GB/T 35892-2018) and were approved by the Committee on Experimental Animal Management of the Chinese Academy of Agricultural Sciences (Beijing) (reference No. 39/15.07.2023). We purchased 30 Kunming mice (specific pathogen-free grade, 3-week-old male) from the Experimental Animal Center of the Inner Mongolia Medical University (Hohhot, China). After a week of acclimatization, the mice were randomly allocated into three groups (n = 10 per group), and each group was fed an experimental diet: a basal diet containing (1) no additive (CK), (2) low-dose (300 mg/kg) UP (UPL), and (3) high-dose (600 mg/kg) UP (UPH) for 28 days. The diet’s composition and nutrient levels are shown in Supplementary Table S1. Each mouse was housed individually in separate ventilated cages under constant conditions (12-h light/dark cycle, 23 °C ± 1 °C). The body weight, feed intake, and diet residue of each mouse were monitored daily. Average daily body weight gain (ADG), average daily feed intake (ADFI), and the ratio of feed to gain (*F*/*G*) were calculated. By the end of the trial, the mice were starved for 12 h and then euthanized for blood collection and other tissue sampling. Serum samples was separated by centrifugation at 3,000 *g* for 15 min and stored at −80 °C until analysis. The levels of alanine aminotransferase (ALT), aspartate aminotransferase (AST), high-density lipoprotein cholesterol (HDL-C), low-density lipoprotein cholesterol (LDL-C), triglyceride (TG), and total cholesterol (TC) in the serum were measured by automatic analyzer (HITACHI 747, Tokyo, Japan).

### Analysis of antioxidant indices in serum and liver

The frozen liver tissue was minced and homogenized (10% w/v) in normal saline, and then the grinding liquid was centrifuged at 3,000 *g* for 10 min at 4 °C. The supernatant and serum thus obtained were used to measure the level of total antioxidant capacity (T-AOC), catalase (CAT), superoxide dismutase (SOD), glutathione peroxidase (GSH-PX), and malondialdehyde (MDA). Antioxidant indices and protein were assessed by commercially available kits from the Jiancheng Bioengineering Institute (Nanjing, China) in accordance with the manufacturer’s instructions.

### Histopathological examination

Fresh liver and colon samples were resected, rinsed with saline, and fixed in 4% paraformaldehyde. The tissues were then embedded in paraffin, sectioned, and subjected to hematoxylin and eosin (H&E) staining. Representative stained sections were selected, and histological images were captured by microscope (Olympus BX51, Japan).

### 16s rRNA gene sequencing and SCFA analysis

Genomic DNA was extracted from colon contents using the EZNA®Soil NDA Kit (Omega Bio-Tek, Inc., USA) following kit protocols. The quality of isolated DNA was measured using a NanoDrop NC2000 spectrophotometer (Thermo Scientific, Waltham, MA, USA) and 1% agarose gel electrophoresis. The DNA obtained was amplified with specific bacterial primers targeting the V3–V4 region of the bacterial 16S rRNA gene using the forward (5′-ACT​CCT​ACG​GGA​GGC​AGC​A-3′) and reverse primers (5′-GGACTACHVGGGTWTCTAAT-3′), and amplicon sequencing using paired-end sequencing (Novaseq-PE250) on the Illumina platform were performed at Gene *Denovo* Biotechnology Co. (Guangzhou, China). The PCR reaction program and analyses of alpha (Chao1, ACE, Shannon, Simpson, and Pielou) and beta bacterial diversities referred to our previous research (Jize et al., 2022). PICRUSt2 analysis was employed to predict functional shifts in the microbiota across different samples. This prediction was based on the level 2 pathways within the Kyoto Encyclopedia of Genes and Genomes (KEGG) database. Further details regarding the PICRUSt2 methodology can be found at https://github.com/picrust/picrust2. We dissolved 200 mg colon contents in 1 mL of ultrapure water and vortexed it, then the SCFA profiles of colon content homogenates were determined using gas chromatography (Agilent 6850, Agilent Technologies Inc., Santa Clara, CA, USA) (Liang et al., 2023).

### Untargeted metabolomics of serum samples

Frozen serum samples (−80 °C) were thawed and vortexed. A 50-μL aliquot was mixed with 300 μL of extraction reagent (acetonitrile: methanol, 1:4 v/v) containing internal standards, vortexed for 3 min, and centrifuged (4 °C, 10 min, 21,380 *g*). The supernatant was chilled (−20 °C, 30 min), recentrifuged, and filtered (0.22-μm membrane) prior to LC-MS analysis. LC-MS analysis was performed using an Agilent 1,290 Infinity II LC system coupled with an Agilent 6550 QTOF mass spectrometer. Analytes were eluted on a Waters ACQITY UPLC HSS T3 C18 column (1.8 μm, 2.1 mm × 100 mm) with a mobile phrase of water (A) and acetonitrile (B), both with 0.1% formic acid, using the following gradient: 0–11 min, 95%–10%, 11–12 min, 10% A; 12–12.1 min, 10%–95% A; 12.1–14 min, 95% A. The column temperature was 40 °C, flow rate 0.40 mL/min, and injection volume 2 μL. Mass spectrometry parameters included ion source voltages of 2.5 kV (positive) and 1.5 kV (negative); gas flow, 8 L/min; fragmentation voltage, 0.135 kV; gas temperature, 325 °C; sheath temperature, 325 °C; sheath flow, 11 L/min; nebulizer, 40 V. Raw MS data was converted to mzXML format (ProteoWizard) and processed with XCMS for retention time correction, peak extraction, and alignment. Principal component analysis (PCA), supervised orthogonal partial least-squares discriminant analysis (OPLS-DA), and hierarchical cluster analysis (HCA) were performed using MetaboAnalyst 6.0 online software (https://www.metaboanalyst.ca). Prior to these multivariate analyses, variables underwent normalization by constant sum, Log_10_ transformation, and autoscaling. Tentative feature identification was achieved by matching accurate mass (mass tolerance <0.01 Da) and MS/MS data (mass tolerance <0.02 Da) against databases (HMDB, MassBank, and the in-house metabolite standard library of Shanghai Bioprofile Biotechnology Co., Ltd.). Only features with fully matched MS/MS information in the database were reported.

### Statistical analysis

Data were expressed as means ± standard deviation (SD). Data analysis was performed using SPSS 20.0 (SPSS, Chicago, IL, USA). The two-tailed Student’s *t*-test was employed for comparisons between two groups, while one-way analysis of variance (ANOVA) followed by Duncan’s *post hoc* test was used for comparisons among three groups. Statistical significance was set at *P* < 0.05. The correlation between gut microbiota and antioxidant function was assessed using Pearson’s correlation analysis.

## Results

### Effects of UP on growth performance and serum biochemical profiles

**FIGURE 1:**
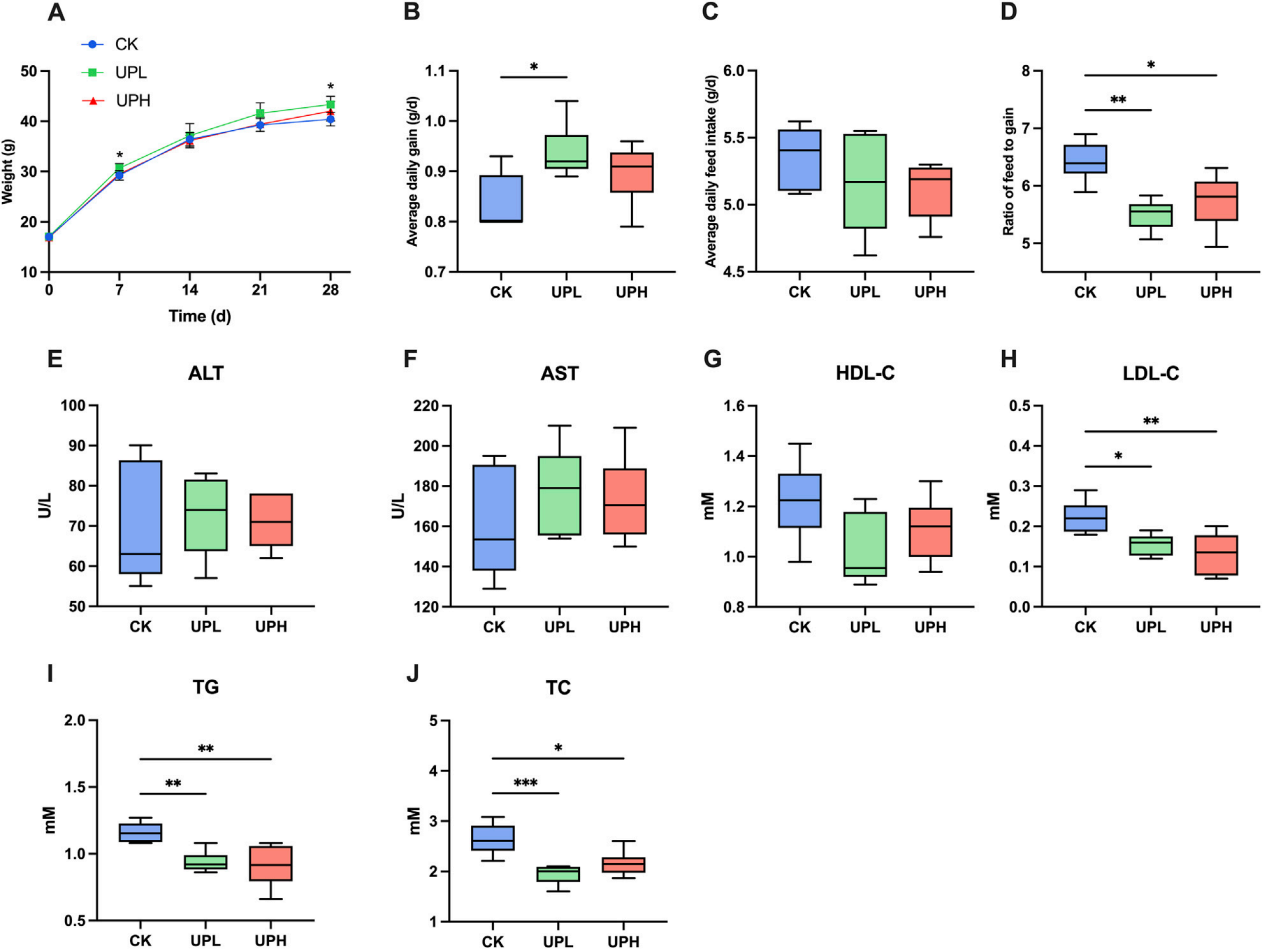
Effects of *Urtica cannabina* polysaccharide (UP) supplementation on growth performance and serum biochemical parameters in mice. **(A)** Body weight of mice (n = 10). **(B)** Average daily gain (ADG) of mice (n = 10). **(C)** Average daily feed intake (ADFI) of mice (n = 10.; **(D)** Ratio of feed to gain (*F/G*) of mice (n = 10). **(E–F)** Liver function (n = 6). **(G–J)** Lipid metabolism (n = 6). ^*^ *P* < 0.05, ^**^ *P* < 0.01. CK, control; UPL, low dose of *U. cannabina* polysaccharide; UPH, high dose of *U. cannabina* polysaccharide; ALT, alanine aminotransferase; AST, aspartate aminotransferase; HDL-C, high-density lipoprotein cholesterol; LDL-C, low-density lipoprotein cholesterol; TG, triglyceride; TC, total cholesterol.

Over the 4-week feeding period, all treatments resulted in a notable increase in the body weight of mice (Figure 1A). Specifically, there were significant differences in body weight on the second week and by the end of the experiment between CK and UPL supplemented groups (*P* < 0.05). Furthermore, as shown in Figures 1B ,D, UPL treatment significantly improved ADG and *F/G*. However, the effect of UP on the ADFI was not detected (*P* > 0.05) (Figure 1C). The effect of UP on the severity of liver injury or liver disease was evaluated by determining AST and ALT activities in serum. As shown in Figures 1E ,F, AST and ALT did not exhibit significant differences among groups, which indicated that the liver metabolic function was relatively favorable and belonged to a health state. Moreover, the serum levels of TG, TC, and LDL-C were significantly decreased (*P* < 0.05) after UP administration compared with those in the CK group (Figures 1H–J). There were no differences in HDL-C levels among the three groups (Figure 1G).

### Effects of UP on antioxidant activities in the serum and liver

**FIGURE 2:**
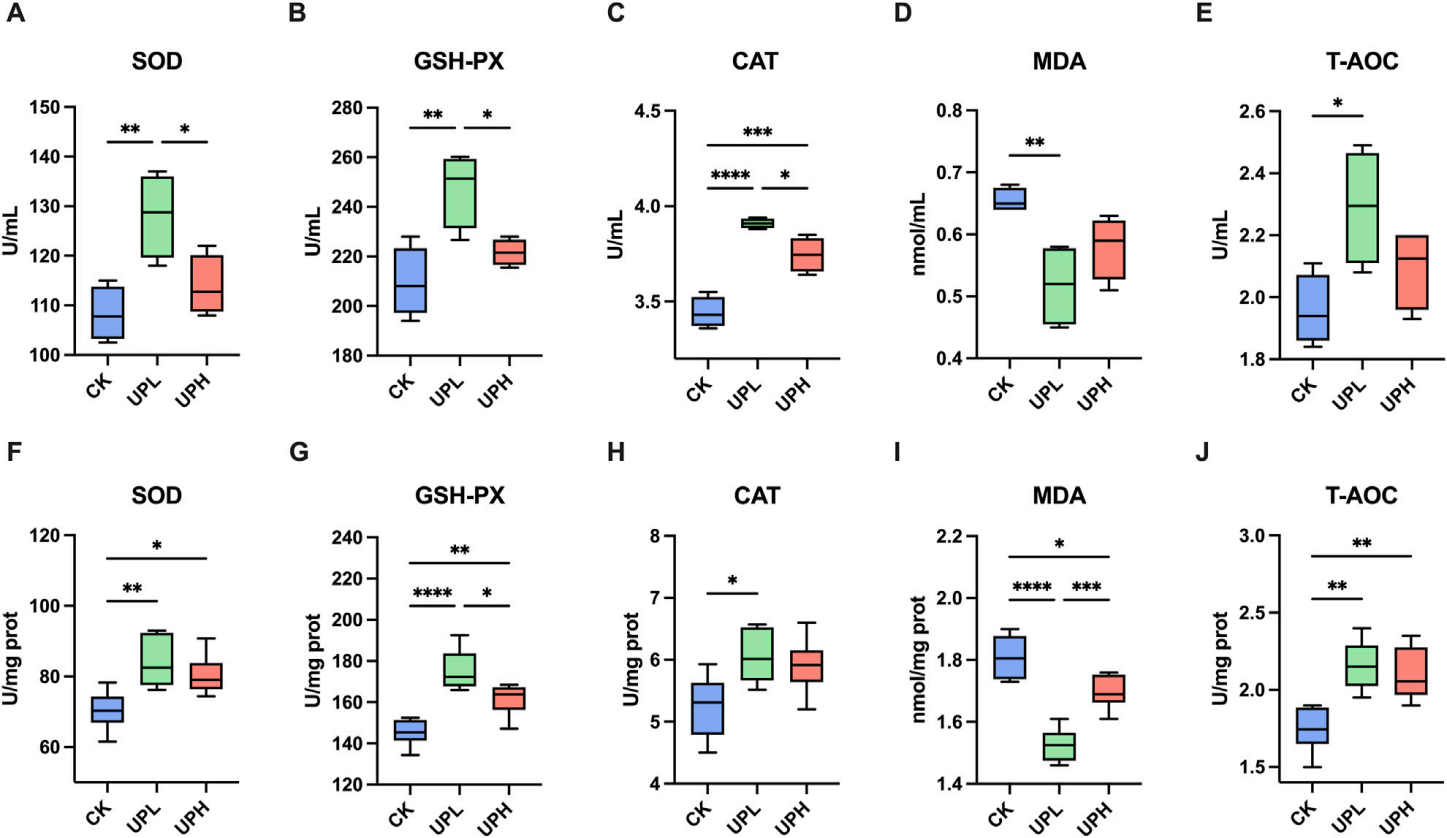
Effects of *Urtica cannabina* polysaccharide (UP) supplementation on serum **(A–D)** and liver **(F–J)** antioxidative activities (n = 6). ^*^ *P* < 0.05, ^**^ *P* < 0.01, ^***^ *P* < 0.001, and ^****^ *P* < 0.0001 CK, control; UPL, low dose of *U. cannabina* polysaccharide; UPH, high dose of *U. cannabina* polysaccharide; SOD, superoxide dismutase; GSH-PX, glutathione peroxidase; CAT, catalase; MDA, malondialdehyde; T-AOC, total antioxidant capacity.

In serum, the level of MDA was notably decreased in mice supplied with low-dose UP (300 mg/kg) in comparison to the CK group (Figure 2D). Meanwhile, low-dose UP supplementation significantly increased the activities of SOD, GSH-PX, CAT, and T-AOC (*P* < 0.05) (Figures 2A–C,E). However, high-dose UP had almost no significant improvement on serum antioxidant capacity compared to the CK group, except for CAT activity. The activities of antioxidant enzymes in liver are shown in Figures 2F–J. Compared to those of the CK group, the SOD, GSH-PX, and T-AOC activities of both UP treatments were enhanced, and MDA content was reduced due to UP supplementation (*P* < 0.05). However, significant upregulated CAT activity was only observed in the UPL group (*P* < 0.05). These results reveal that low doses of UP addition in mice exhibited optimal antioxidant capacity in comparison to the high-dose UP treatment.

### Histopathological analysis

**FIGURE 3:**
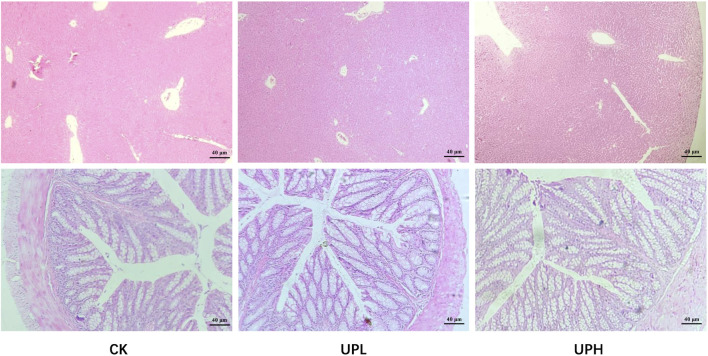
Effects of *Urtica cannabina* polysaccharide (UP) supplementation on the histological structure of liver and colon (n = 4). Representative images were captured by a microscope at 40×. CK, control; UPL, low dose of *U. cannabina* polysaccharide; UPH, high dose of *U. cannabina* polysaccharide.

We then investigated the effects of UP treatments on liver and colon histology. As shown in Figure 3, UP treatments caused no adverse impact on the liver microstructure. Hepatocytes in all groups remained radially organized around the central vein, displaying clear cell boundaries and no visible lipid droplets. Likewise, H&E staining of the colon revealed well-organized tissue architecture in both the CK and UP-intervention groups, with an intact mucosal epithelium and no signs of inflammatory cell infiltration.

### Effects of UP on the composition and function of gut microbiota

**FIGURE 4:**
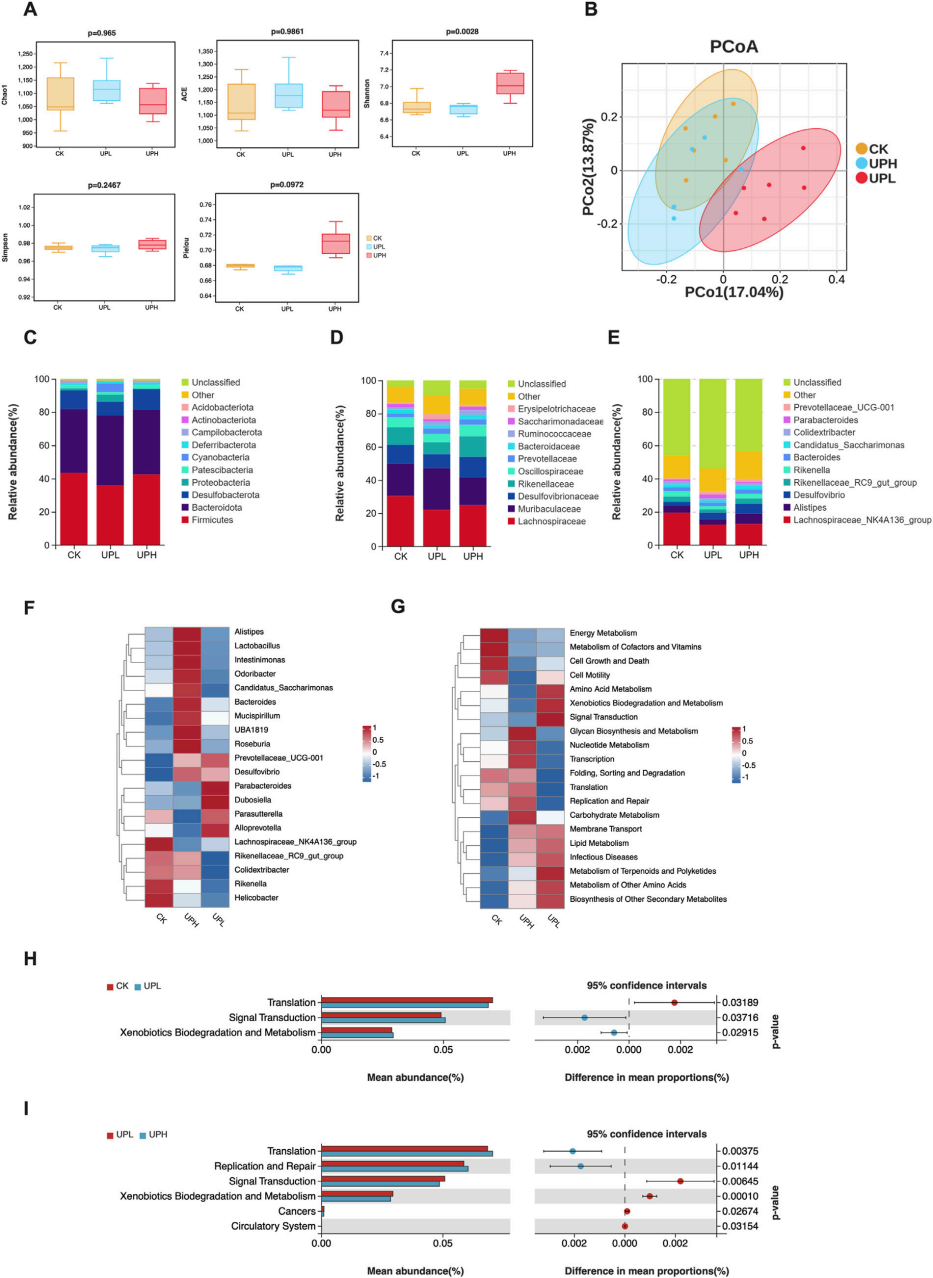
Effects of *Urtica cannabina* polysaccharide (UP) supplementation on the structure and function of colon contents microbial community (n = 6). **(A)** α-diversity, including richness index (Chao1 and ACE), bacterial diversity index (Shannon and Simpson), and evenness index (Pielou). **(B)** Principal coordinate analysis (PCoA) of the overall colon contents microbiota in mice based on unweighted UniFrac distance. **(C–E)** Comparisons of gut bacterial communities in mice under different dietary treatments. The microbiota composition at the phylum **(C)**, family **(D)**, and genus **(E)** levels. **(F)** Heatmap of top 20 bacterial genera of all groups. **(G)** Heatmap of top 20 gene families based on PICRUSt2 function prediction from gut microbiota. **(H)** Metabolic pathways with significant differences between CK and UPL groups. **(I)** Metabolic pathways with significant differences between UPL and UPH groups. CK, control; UPL, low dose of *U. cannabina* polysaccharide; UPH, high dose of *U. cannabina* polysaccharide.

As shown in Figure 4A, except for the Shannon index, most α-diversity indices exhibited no significant differences between groups, suggesting that UP administration may have a limited effect on the diversity and richness of the murine gut microbiota. Furthermore, we assessed whether UP intervention altered the overall gut microbial community structure using β-diversity analysis. The results revealed that UP diet caused a significant separation of gut microbiota between the CK and UPL supplementation groups, but not the UPH group (Figure 4B).

Reflecting the β-diversity variations, UP impacted the relative abundance of bacteria across different taxonomic levels. The bacterial communities at the phylum level in all groups were primarily represented by Firmicutes, Proteobacteria, Bacteroidetes, and Deferribacteres, which together constituted over 86% (Figure 4C). At the family level, in primary microbiota (relative abundance >1%), UP administration remarkably decreased the abundance of Lachnospiraceae (Figure 4D). Lower relative abundance of Desulfovibrionaceae was only observed in the UPL group compared to the CK group. In contrast, the UP groups exhibited a higher relative abundance of Ruminococcaceae than the CK group. At the genus level, the top ranked bacterial genera of all groups were compared (Figure 4E), and bacterial genera with relative abundances below 0.1% were classified as others. We found that UP supplementation reduced the relative abundance of Lachnospiraceae_*NK4A136_group*. Compared to the CK group, low doses of UP significantly decreased the abundance of *Mycoplasma* (Supplementary Figure S1), while high doses of UP significantly increased the abundance of *Roseburia* (Supplementary Figure S1). Moreover, the relative abundances of *Parabacteroides* (*P* = 0.0973) and *Dubosiella* (*P* = 0.0648) tended to increase in the UPL treatment group (Supplementary Figure S1). To visualize the distribution of microbial communities among three groups, the relative abundance of 20 genera are displayed using a heatmap (Figure 4F). Some bacterial genera exhibited remarkable differences in relative abundances between groups. For example, *Alistipes*, *Lactobacillus*, *Intestinimonas*, *Odoribacter*, *Candidatus_Saccharimonas*, *Bacteroides*, *Mucispirillum*, *UBA 1819*, and *Roseburia* were enriched in the UPH group. However, low doses of UP increased the abundance of *Parabacteroides*, *Dubosiella*, *Parasutterella*, and *Alloprevotella*. These results imply that UPL and UPH treatments modulate the gut microbiota in distinct ways. In summary, low doses of UP seem to induce a potentially favorable gut microbiota profile and enhance the relative abundance of probiotic bacteria.

Furthermore, a heatmap depicting the distribution of the top 20 predicted functional metabolic pathways is shown in Figure 4G. Consistent with the distribution of microbial communities, the functional metabolic pathways also exhibited distinct variation among the three groups. Specifically, signal transduction and xenobiotics biodegradation and metabolism were enriched in the UPL group compared with the CK and UPH groups (*P* < 0.05) (Figures 4H,I).

### Effects of UP on SCFAs

**FIGURE 5:**
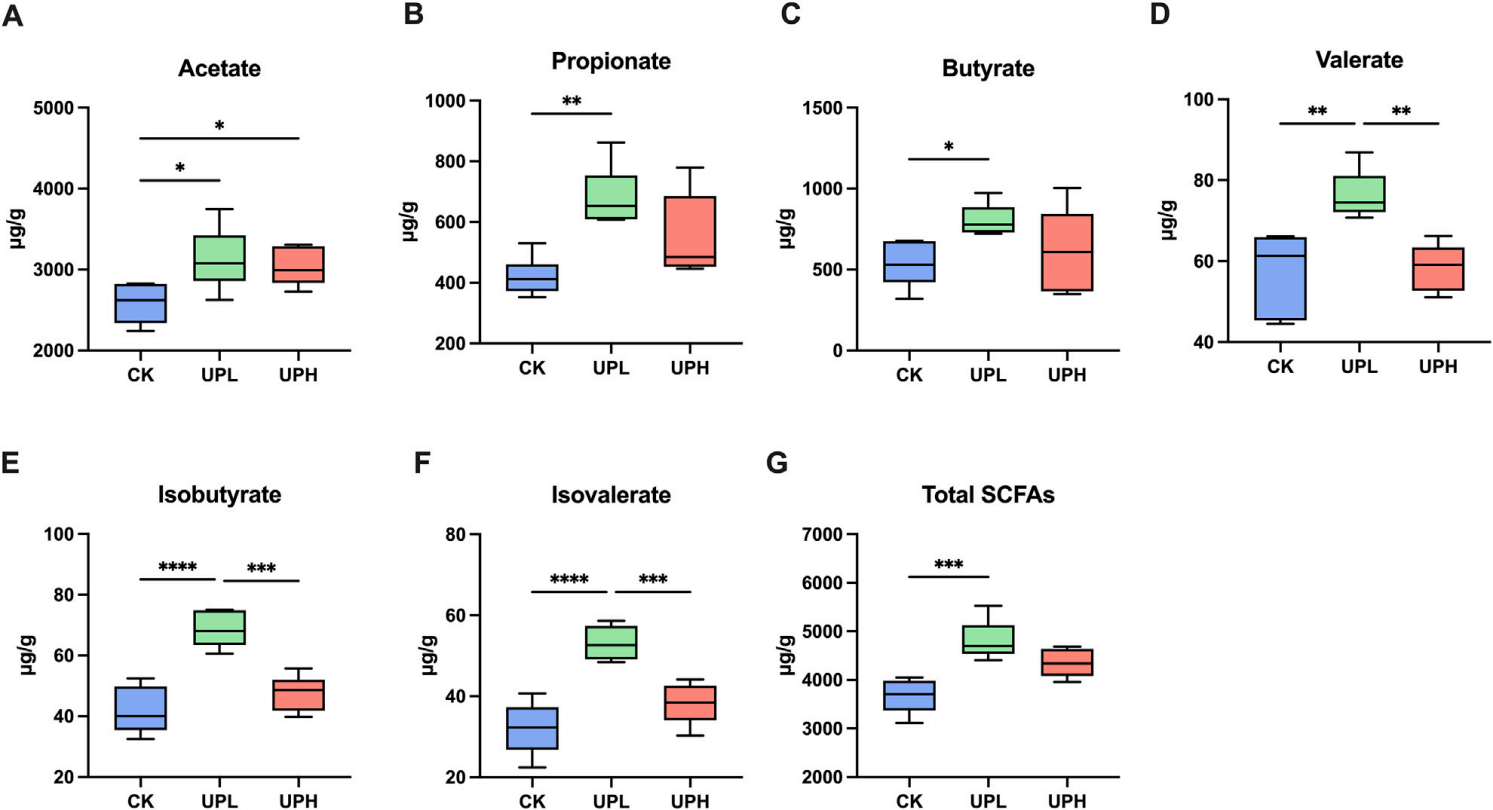
Effects of *Urtica cannabina* polysaccharide (UP) supplementation on the short-chain fatty acids (SCFAs) **(A-G)** concentrations of colon contents. Data are presented as means ± SD (n = 6). ^*^ *P* < 0.05, ^**^ *P* < 0.01, ^***^ *P* < 0.001, and ^****^ *P* < 0.0001. CK, control; UPL, low dose of *U. cannabina* polysaccharide; UPH, high dose of *U. cannabina* polysaccharide.

The SCFA concentrations in colon contents are presented in Figure 5. Acetate, propionate, and butyrate as primary SCFA components were detected in all groups. As shown in Figures 5A–F, the UPL group exhibited significantly elevated total SCFA levels compared to the CK group. Inter-group analysis revealed that UP selectively increased acetate concentration in colon contents (*P* < 0.05) relative to the CK group, irrespective of the administrated dose (Figure 5A). The mean acetate levels in the CK, UPL, and UPH groups were 2,584, 3,130, and 3,028 μg/g, respectively. In colon contents, no significant differences were observed between the CK and UPH groups for all SACFs other than acetate.

### Effects of UP on serum metabolites

**FIGURE 6:**
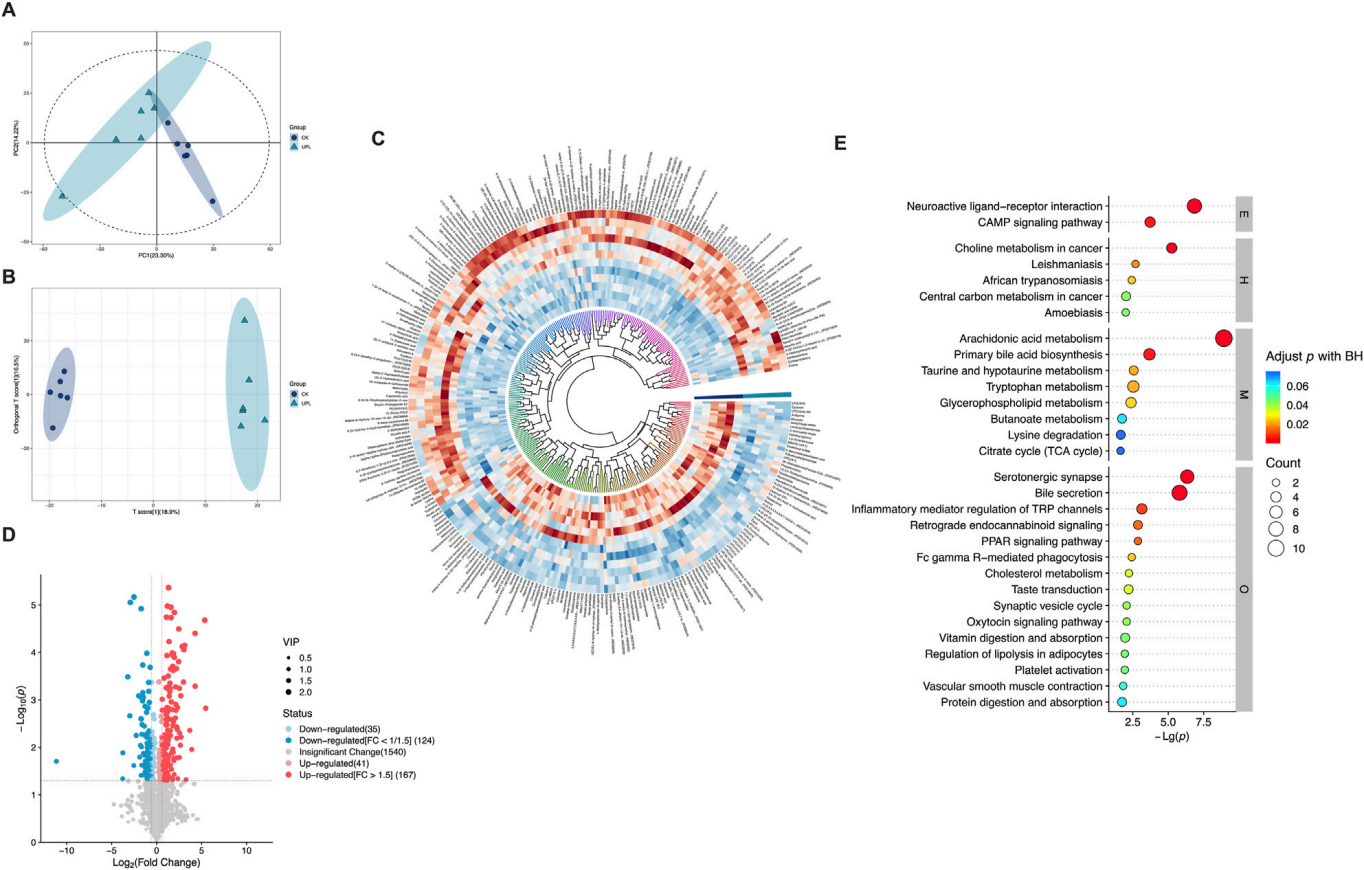
Comprehensive analysis of untargeted serum metabolomics of mice in the CK (n = 6) and UPL (n = 6) groups. **(A,B)** PCA (A) and OPLS-DA plots of metabolomics data. **(C)** Classification and expression changes of differential metabolites in two groups by circle heatmap. **(D)** Volcano map of metabolites. Horizontal axis represents logarithm of the relative content fold change: Log_2_(Fold Change). Vertical axis represents level of significant difference: Log_10_ *P*-value; size of the dot represents VIP value. **(E)** Top 30 enriched KEGG pathways (level 1) of differentially expressed metabolites. Horizontal axis represents level of significant difference: log_10_ *P*-value; size of the dot represents number of metabolites. CK, control; UPL, low dose of *Urtica cannabina* polysaccharide; E, environmental information processing; H, human diseases; M, metabolism; O, organismal systems.

**FIGURE 7:**
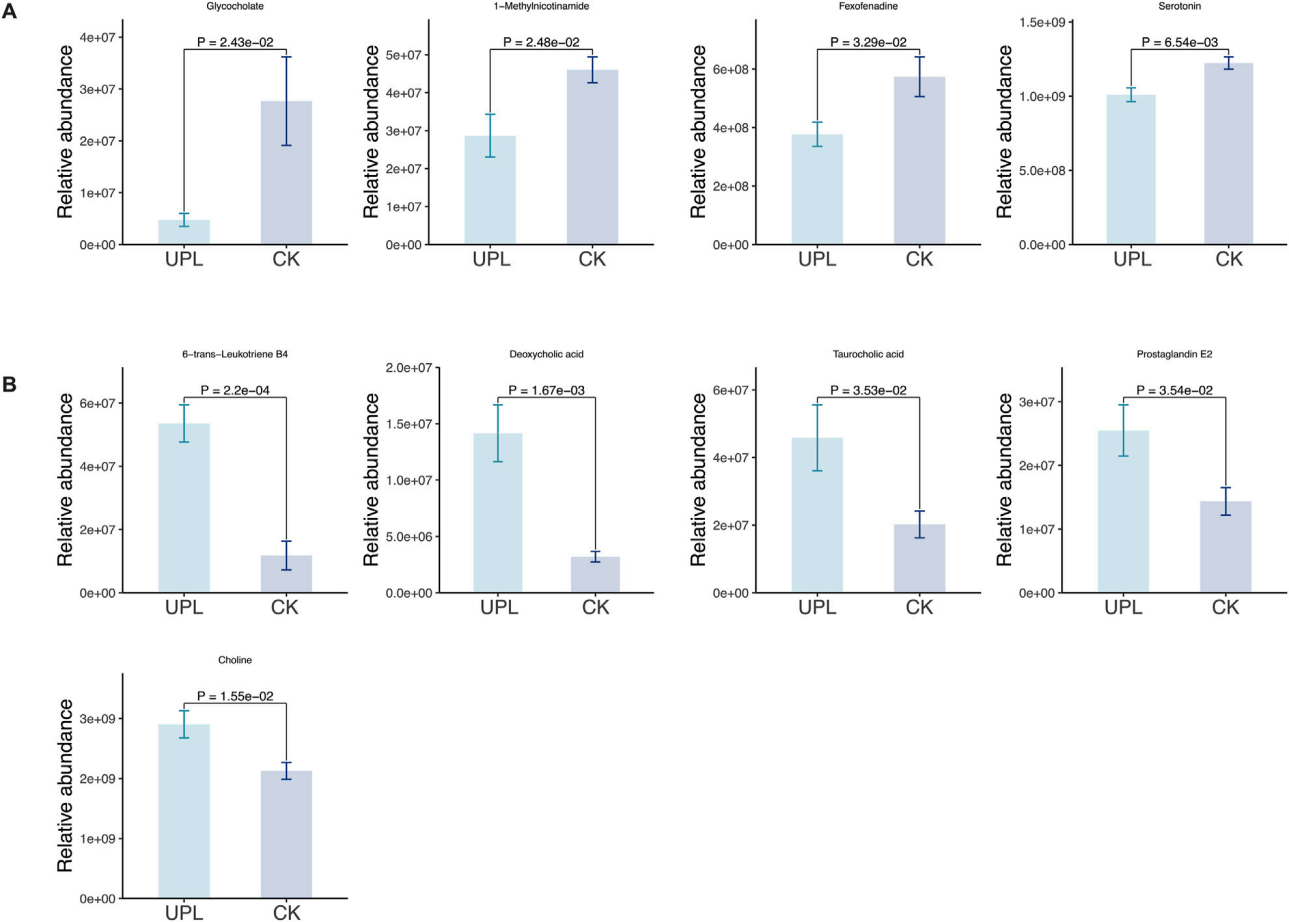
Effects of *Urtica cannabina* polysaccharide (UP) supplementation on serum metabolites of the bile secretion pathway in CK and UPL groups. **(A)** Bar chart showing ion intensities of downregulated metabolites. **(B)** Bar chart showing ion intensities of upregulated metabolites. CK, control; UPL, low dose of *Urtica* polysaccharide.

Functioning as phenotype regulators, metabolites were profiled via untargeted metabolism in mouse serum to explore alternations in host metabolism between the CK and UPL groups. Across all serum samples, a total of 1907 metabolites were detected (908 in positive and 999 in negative ion modes) (Supplementary Table S2), which were then subjected to multivariate statistical analyses. The PCA analysis of these metabolites (Figure 6A) clearly separated serum samples from the CK and UPL groups into distinct clusters. A similar distinct separation was observed in the OPLS-DA score scatter plot (Figure 6B). OPLS-DA, a supervised discriminant analysis statistical method, more effectively highlights inter-sample differences than PCA. Differentially expressed metabolites were identified using OPLS-DA-model-derived variable importance projection (VIP) scores ≥1.0, *P* < 0.05 in Student’s t*-*test as well as fold change (FC) values ≥1.5 or ≤1/1.5. This analysis revealed 291 metabolites (167 upregulated and 124 downregulated) with significant abundance differences between the CK and UPL groups (Figures 6C,D). The most abundant metabolite classifications were lipids and lipid-like molecules (50.45%), organoheterocyclic compounds (13.74%), organic acids and derivatives (13.13%), benzenoids (8.96%), and phenylpropanoids and polyketides (5.37%). The top 30 enriched KEGG pathways based on these differential metabolites are presented in Figure 6E, with arachidonic acid metabolism, bile secretion, neuroactive ligand–receptor interaction (NLRI), and serotonergic synapse exhibiting the highest number of associated metabolites (over five metabolites). Figure 7 further details the abundance of bile secretion pathway-related metabolites across the two groups. These metabolites are closely related to hepatic function, with four being downregulated and five upregulated.

### Analysis of the correlations between gut microbiota and antioxidant function

**FIGURE 8:**
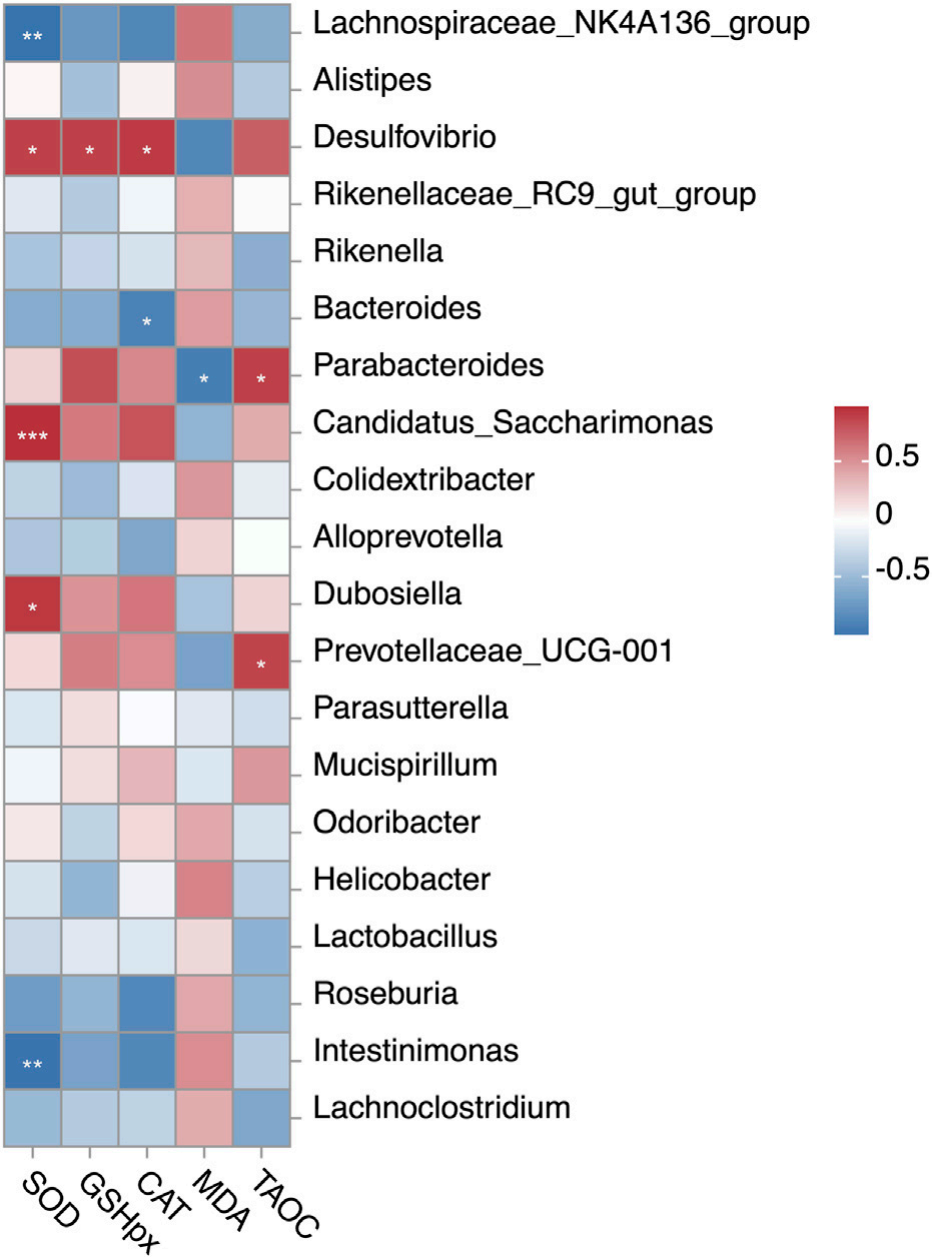
Correlation between the relative abundances of gut bacteria and hepatic antioxidant indexes. Pearson’s correlation coefficients (*r*) are given, with *r* < 0 indicating negative correlation (blue), *r* = 0 indicating no correlation (white), and *r* > 0 indicating positive correlation (red). “*”, “**”, and “***” indicate significance level at 0.05, 0.01 and 0.001, respectively. SOD, superoxide dismutase; GSH-PX, glutathione peroxidase; CAT, catalase; MDA, malondialdehyde; T-AOC, total antioxidant capacity.

To determine whether UP-induced changes in the hepatic antioxidant function were associated with the effects on gut microbiota, we performed Pearson’s correlation coefficient (*r*) analysis (Figure 8). UPL-induced shifts in *Parabacteroides* were positively linked to T-AOC activity and negatively associated with MDA level. *Dubosiella* enriched in the UPL supplemented mice showed positive correlation with SOD activity. *Candidatus_Saccharimonas*, a genus with higher abundance in the UPH group, was positively associated with SOD activity. Additionally, we found that higher abundances of *Intestinimonas* and *Bacteroides* had negative correlations with SOD and CAT activities, respectively.

## Discussion

Polysaccharides are primary bioactive constituents of *Urtica cannabina*. While numerous studies have highlighted the metabolic benefits of other key bioactive constituents from *Urtica* species, such as phenolics and flavonoids, in ameliorating oxidative and inflammatory states associated with metabolic syndrome (Bhusal et al., 2022; Carvalho et al., 2017), the physiological effects of its polysaccharides are less documented. Furthermore, most research on the bioactive constituents of *U. cannabina* has focused on interventions in sub-healthy or diseased individuals (Samakar et al., 2022), with limited information available on their impact on healthy subjects. This study aimed to fill this gap by investigating how different doses of UP affect metabolic phenotypes, serum metabolites, gut microbiota compositions, and SCFA profiles in healthy mice, thereby exploring the associations between host metabolism and the gut microbiome.

Our results demonstrated that UP supplementation, particularly at a low dose of 300 mg/kg, enhanced growth performance, as evidenced by improved ADG and *F/G.* This finding aligns with previous research demonstrating that plant polysaccharides from *Artemisia argyi* and *A. ordosica* L. improved growth performance in broilers and rats, respectively (Xing et al., 2020; Zhang J et al., 2016), reinforcing the growth-promoting potential of polysaccharide as a major bioactive component in plant aqueous extracts. Beyond growth, UP administration in the current study also significantly influenced serum lipid profiles by reducing TC, TG, and LDL-C. Hyperlipidemia, a lipid metabolism disorder characterized by elevated TC, TG or LDL-C, or reduced HDL-C, is a major risk factor associated with atherosclerosis (Chai et al., 2022) and often co-occurs with other cardiovascular risks like diabetes and hypertension (Bragg and Walling, 2015). Our observations are consistent with prior *in vivo* studies where aqueous extracts of nettle demonstrated combined hypoglycemic and hypolipidemic activities (Mehran et al., 2015). Specifically, a noticeable hypolipidemic effect of nettle aqueous extract (150 mg/kg/day) was reported in rats on a high-fat diet, manifested as a decrease in serum TC and LDL-C levels and in the LDL-C/HDL-C ratio (Daher et al., 2006).

Many chronic diseases, such as obesity, diabetes, and cardiovascular diseases, are strongly linked to *in vivo* oxidative stress resulting from the overproduction of reactive oxygen species (ROS) (Zhang P et al., 2016). Natural polysaccharides exhibit broad therapeutic applications in treating these conditions, primarily due to their potent antioxidant properties mediated by the regulation of signal transduction pathways, activating enzymes, and scavenging free radicals (Wang et al., 2017; Xing et al., 2020). In this study, UP supplementation, particularly at low doses (UPL), enhanced both hepatic and serum antioxidant capacity, evidenced by reduced MDA levels and concurrently elevated activities of SOD, GSH-PX, CAT, and T-AOC. H&E staining revealed no significant histological changes in the liver following UP treatments, indicating that UP preserved normal hepatocyte function. This was consistent with the lack of significant differences in serum ALT and AST levels between the CK and UP groups, as these are commonly used indicators of liver function.

Given the established role of the gut microbiome as a critical modulator of system metabolism (Flint et al., 2012), we further investigated its potential contribution to these potent antioxidant effects. We hypothesized that the benefits observed in the liver and serum were possibly mediated through the gut–liver axis, a crucial pathway of bidirectional communication, where gut microbial metabolites influence liver function and bile acids (BAs), in turn shaping the microbial community (Foley et al., 2019; Wang et al., 2025a). The results obtained support this hypothesis, revealing that UP supplementation significantly modulated the gut microbiota composition. Specifically, the abundances of *Parabacteroides* and *Dubosiella* were enriched in the UPL group, showing a significant positive correlation with hepatic antioxidant capability in mice. *Parabacteroides*, a potential probiotic prevalent in the gastrointestinal tract of humans and animals, is known for producing secondary BAs and acetate (Lei et al., 2021; Wu, 2023). Dietary *Parabacteroides* intervention has been proven to enhance antioxidant capacity and reduce oxidative stress-induced tissue damage by activating the Nrf2 antioxidant response pathway in piglets (Wu et al., 2024). Similarly, *Dubosiella* has demonstrated to the ability to boost secondary BA metabolism in mice following probiotic intervention, leading to ameliorated inflammation and improved intestinal barrier function (Liu et al., 2025). Extensive literature also supports the crucial role of *Dubosiella* in reducing oxidative stress and tissue inflammation, primarily by increasing SCFA production—particularly butyrate (Wei et al., 2023; Ye et al., 2023). Further evidence for increased microbial metabolic activity in the UPL group was provided by the KEGG function prediction using PICRUSt2 analysis (Figures 5H,I), which predicted an enrichment of pathways involved in signal transduction and xenobiotic biodegradation and metabolism. These findings indicate a greater potential for the gut microbiota to modify compounds such as primary BAs and generate signaling molecules that interact with the host through the gut–liver axis.

Consistent with the enrichment of these microbes, UPL treatment significantly increased the concentrations of total and individual SCFAs in gut contents, an effect not observed with the UPH treatment. These SCFAs can traverse the gut–liver axis and exert beneficial effects on hepatic functions (Wang et al., 2025b). Furthermore, SCFAs produced by gut microbiota contribute to numerous health benefits, including maintaining gut barrier function, supplying intestinal energy, regulating immunity, and exhibiting antioxidant, anti-inflammatory, and anti-tumor properties (Dalile et al., 2019). SCFAs modulate host redox homeostasis by either activating the Nrf2 signaling pathway, which increases the expression of antioxidant enzymes (SOD and CAT), or by inhibiting NADPH oxidase 2 (NOX2) activity, thereby reducing ROS production (Wang et al., 2025a). Collectively, these findings indicate that UP supplementation positively affects host antioxidant function, likely mediated by its modulation of the gut microbiome and subsequently production of beneficial metabolites like SCFAs and secondary BAs.

Hence, we performed untargeted serum metabolomics to identify differential metabolites that may be responsible for the antioxidant benefits of UP intervention. A clear separation between CK and UPL groups was observed both in the PCA and OPLS-DA analyses, indicating an effective influence on the metabolism of mice due to UP supplementation. Pathway enrichment analysis of the identified serum metabolites revealed a significant involvement in bile secretion. In addition to their role in emulsifying of dietary fats, BAs act as signaling molecules with important regulatory effects on metabolic homeostasis (Ahmad and Haeusler, 2019; Garruti et al., 2017). Primary and secondary BAs can regulate host immunological and antioxidative processes through the activation of either the nuclear farnesoid X receptor (FXR) or the cell-surface G protein-coupled bile acid receptor 1 (GPBAR1) (Punzo et al., 2024). Notably, deoxycholic acid (DCA), a predominant constituent of secondary BA, and taurocholic acid (TCA), a conjugated primary BA, were significantly enriched in the UPL treatment (Figure 7). DCA is known to act as a signaling molecule to activate the FXR (Xiang et al., 2021). Previous studies have demonstrated that FXR agonists activate the Nrf2 signaling, thereby decreasing the production of ROS and MDA levels and concurrently upregulating the expression of CAT, glutathione S-transferase (GST), and SOD in a diabetic mice model (Li et al., 2020). TCA was demonstrated to have a positive correlation with the expression of BA receptors, especially FXR signaling (Xu et al., 2022). Due to its higher polarity and water solubility, TCA was reported to pass through the cell membrane and exert intracellular antioxidant and anti-inflammatory effects (Ge et al., 2023). Importantly, TCA serves as a substrate for gut microbes to produce DCA by converting taurine and cholic aid (Ridlon et al., 2016). This interaction highlights the crucial role of BAs in the enterohepatic circulation, where they interact dynamically with gut microbiota. The microbiota actively participate in BAs metabolism through processes like oxidation, fermentation, reduction, and transformation. Certain gut microbes modify BAs through hydrolysis or deoxygenation, resulting in the formation of secondary BAs, which possess distinct metabolic pathways and biological functions that may influence liver health.

**FIGURE 9:**
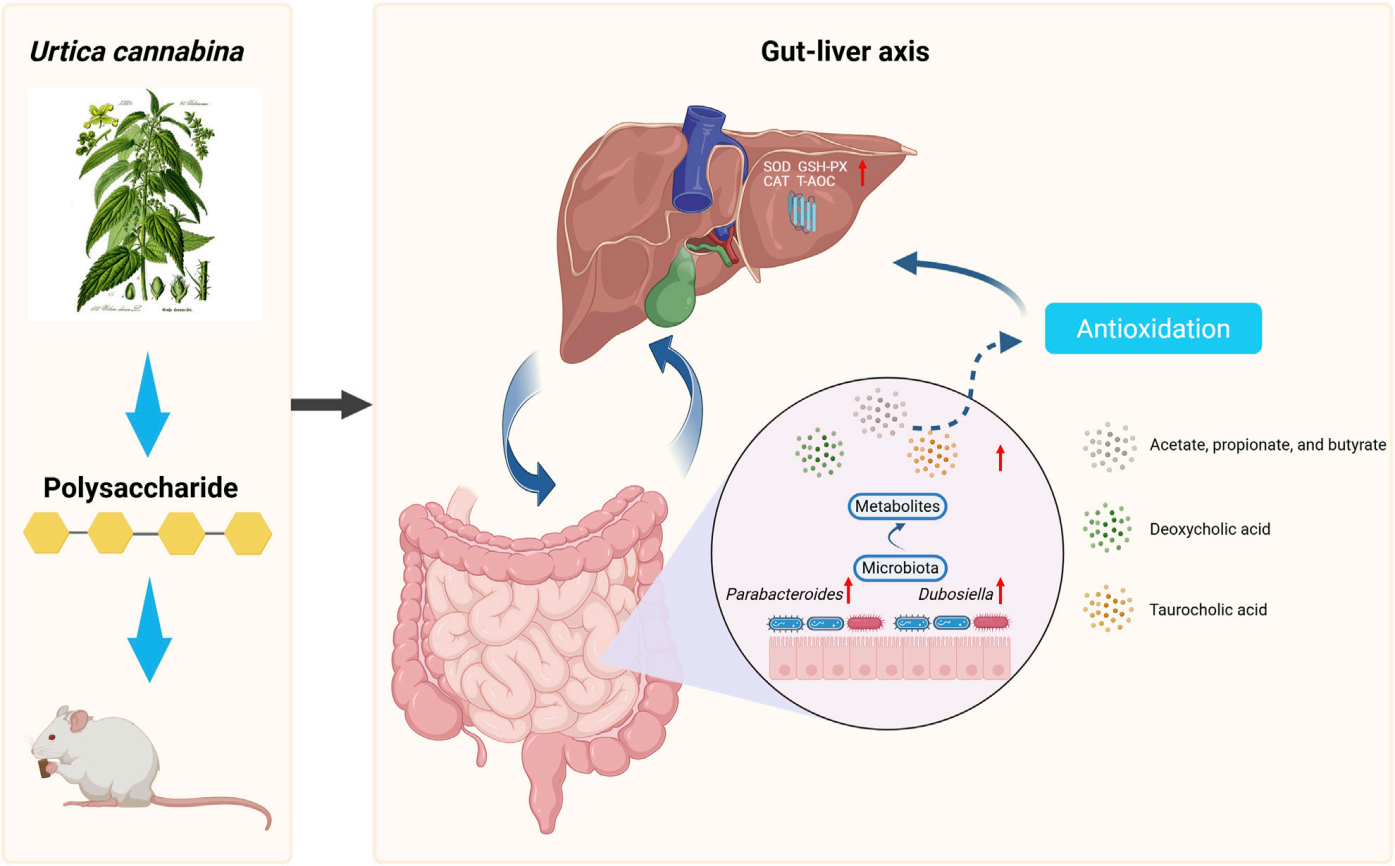
*Urtica cannabina* polysaccharide enhances systemic antioxidant status by modulating the gut–liver through alternations in gut microbiota and regulation of bile acid secretion signaling pathway.

Therefore, we speculate that UPL may improve host antioxidant function through a mechanism involving the gut–liver axis. Specifically, UPL treatment enriches for beneficial gut microbes, including *Parabacteroides* and *Dubosiella*, which in turn increases the production of SCFAs and modulates BA metabolism, leading to elevated levels of signaling molecules like DCA. These microbial metabolites then possibly active hepatic antioxidant pathways, such as FXR/Nrf2, to protect against oxidative stress (Figure 9). However, the current study is still limited by the lack of direct validation of key signaling proteins, inflammatory markers, and portal metabolite flux. The findings were further constrained using healthy animals and the lack of a positive control group, which limits the assessment of therapeutic relevance and comparative efficacy. Future work should confirm pathway activation, clarify the role of specific metabolites such as DCA, and evaluate the efficacy in pathological models using advanced molecular profiling and portal venous sampling to establish causal mechanisms.

## Conclusion

In the search for novel ingredients to meet the growing demand for functional foods, this study identifies UP as a highly promising candidate. Our findings demonstrate that low-dose UP acts as a potent metabolic modulator, enhancing growth, improving lipid profiles, and promoting systemic antioxidant defenses in healthy mice. We propose a novel mechanism operating through the gut–liver axis whereby UP selectively enriches beneficial gut microbes, such as *Parabacteroides* and *Dubosiella*, leading to an increased production of SCFAs and secondary BAs like DCA. These microbial metabolites then likely stimulate hepatic antioxidant pathways. By providing mechanistic insights into the pathway from ingestion to systemic health benefit, our findings strongly support the development of UP as a high-value functional food ingredient for maintaining host metabolic homeostasis and promoting overall wellbeing.

## Data Availability

All data needed to evaluate the conclusions in the paper are presented in the paper and the supplementary information. The datasets generated and/or analyzed during the current study are available in the Sequence Read Archive of the National Center for Biotechnology database repository with accession project number PRJNA1321720. Additional data related to this paper may be requested from the corresponding authors.

## Supplementary material

SUPPLEMENTARY FIGURE S1 Comparison of the bacteria (genus level) within the colon of mice in CK, UPL, and UPH groups.
