# Supplementary figures and images for: Nettle (Urtica cannabina L.) polysaccharides as a novel dietary supplement: enhancing systemic antioxidant status via modulation of the gut–liver axis
Source: Front Pharmacol. 2025 Nov 19;16:1692189. doi: 10.3389/fphar.2025.1692189 (PMC12672437; doi:10.3389/fphar.2025.1692189)

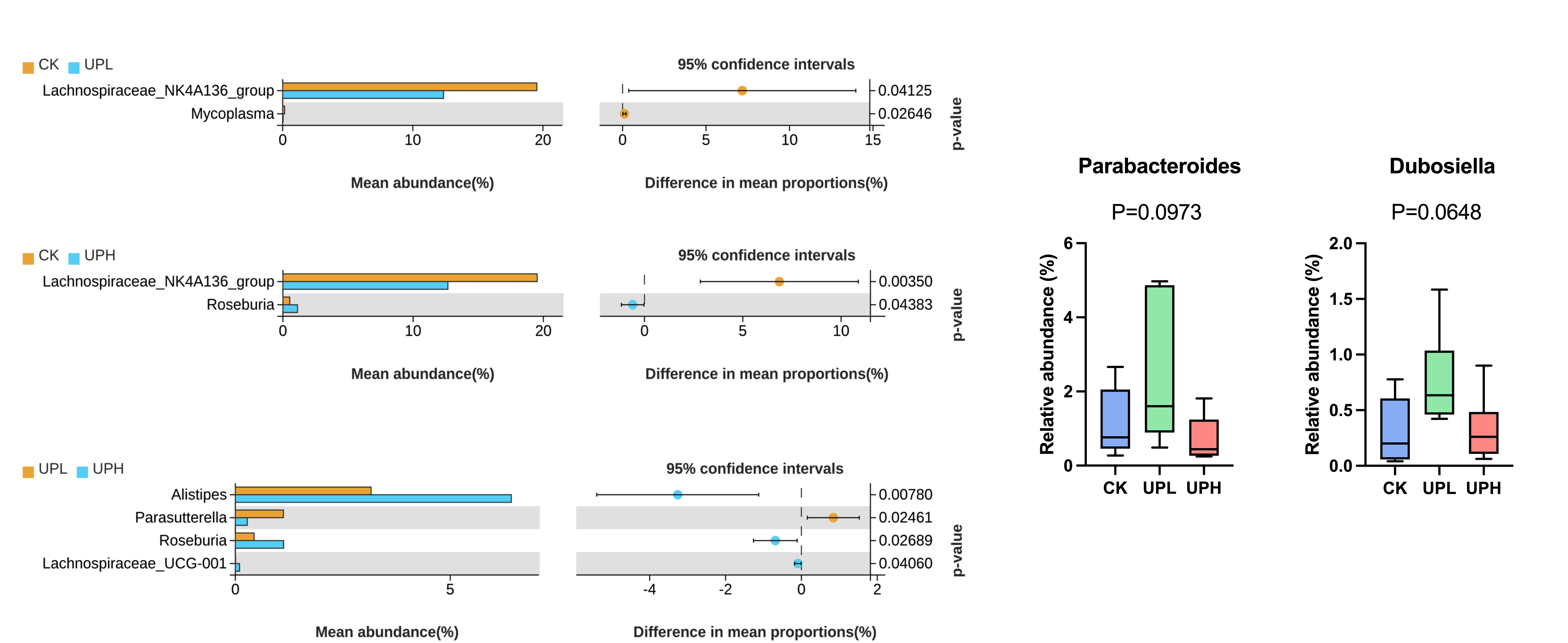

Supplement: Supplementary file 2 [file Image1.tiff]
