# Supplementary material for: Nettle (Urtica cannabina L.) polysaccharides as a novel dietary supplement: enhancing systemic antioxidant status via modulation of the gut–liver axis
Source: Front Pharmacol. 2025 Nov 19;16:1692189. doi: 10.3389/fphar.2025.1692189 (PMC12672437; doi:10.3389/fphar.2025.1692189)
Supplement: Supplementary file 3 [file Table1.docx]

Table S1 Composition and nutrient levels of basal diet for mice (as dry matter basis).

| Items | Basal diet |
| --- | --- |
| Ingredients (%) |  |
| Corn starch | 37.74 |
| Casein | 7.50 |
| Soybean meal | 15.00 |
| Dextrin | 13.20 |
| Saccharose | 10.00 |
| Corn oil | 7.00 |
| Cellulose | 5.00 |
| Mineral premix^1^ | 3.50 |
| Vitamin premix^2^ | 1.00 |
| *L*-Cystine (purity 98%) | 0.30 |
| Choline chloride | 0.25 |
| tert-Butylhydroquinone | 0.01 |
| Total | 100.00 |
| Nutriment level |  |
| Crude protein (%) | 19.60 |
| Crude fat (%) | 6.50 |
| GE (KJ/kg)^3^ | 15.77 |
| Tryptophan (%) | 2.11 |
| Threonine (%) | 6.72 |
| Methionine (%) | 4.61 |
| Lysine (%) | 13.01 |
| Available phosphorus (%) | 0.30 |
| Calcium (%) | 0.54 |

^1^Supplied the following per kilogram of diet: Zn, 38 mg; Fe, 45 mg; Mn, 10 mg; Cu, 6 mg; I, 0.2 mg.

^2^Supplied the following per kilogram of diet: vitamin A, 4000 IU; vitamin D, 1000 IU; vitamin E, 75 IU; vitamin K, 0.9 mg; vitamin B_12_, 0.025 mg; vitamin B_6_, 6 mg; pantothenic acid, 15 mg; vitamin B_2_, 6 mg, folic acid, 2 mg; vitamin B_1_, 5 mg; biotin, 0.2 mg.

^3^The number was calculated value.
